# A phenomenological study of compassion satisfaction among social work educators in higher education

**DOI:** 10.3389/fpsyg.2023.1176786

**Published:** 2023-09-05

**Authors:** Sultan A. Shubair, Ben Miller, Jean Zelenko

**Affiliations:** ^1^Social Studies Department, College of Humanities and Social Sciences, King Saud University, Riyadh, Saudi Arabia; ^2^Raymond A. Kent School of Social Work and Family Science, University of Louisville, Louisville, KY, United States

**Keywords:** compassion satisfaction, self-care, social support, education, higher education

## Abstract

**Background:**

Compassion satisfaction (CS) is a phenomenon that has been studied among the helping professions, such as nursing and social work and has been linked to stress, burnout, compassion fatigue, and vicarious trauma. Social work educators may also experience these same issues, yet more research is needed on how they might counter the negative impacts associated with this type of work by utilizing their experiences of CS.

**Objectives:**

A phenomenological study was carried out to explore and describe how social work educators in higher education experiences CS.

**Methods:**

Eleven in-depth interviews with social work educators were conducted, and constructivist grounded theory techniques were utilized to analyze the data.

**Results:**

Social work educators experienced CS within the education and personal realms, which encompassed four different elements: achievement, support, balance, and empathy.

**Discussion:**

The four elements of CS were utilized by social work educators in this study as coping strategies to enhance their experience of CS, thus encountering threats to CS, such as institutional barriers, interaction with administrators and colleagues, and work overload.

**Conclusion:**

Interventions fostering compassion satisfaction and reducing compassion fatigue, burnout, and stress should be considered, including interventions that increase the sense of accomplishment, promote holistic self-care, encourage administrative and collegial support, and improve work-life balance.

## Introduction

The work demands associated with being an educator in higher education institutions could produce adverse negative outcomes on this population’s professional quality of life. For instance, high levels of time pressure from needing to develop curricula and course materials, teaching, training, and mentoring students, conducting and publishing research, attending conferences, and participating in committee, departmental, and faculty meetings with a lack of administrative and collegial support have been linked to an increased the likelihood of stress and burnout among this population (Hoffman et al., 2007; Kim and Stoner, 2008; Watts and Robertson, 2011; Chen, 2022). The stressors associated with working in higher education environment have also been shown to diminish worker productivity, contribute to high resignation rate, and increase emotional and physical problems, such as anxiety, depression, emotional exhaustion, burnout, and depersonalization, which may lead to poor job satisfaction and compassion fatigue (Watts and Robertson, 2011; Sangganjanavanich and Balkin, 2013; Sabagh et al., 2018; Raimondi, 2019).

The literature has also shown that educators in higher education invest a great amount of their time and effort in caring about their students, particularly distressed ones (^21,33^). Deeply caring for distressed students over time and exercising and empathy, which has been described as the ability to cognitively understand another person’s emotions, express and show concern, feel those emotions, and be prepared to respond properly to the person’s needs (Hatfield et al., 2011; Levett-Jones et al., 2019) have been associated with experiencing stress and becoming more vulnerable to secondary traumatic disorder, neglectful self-care, and compassion fatigue (^42,11^). However, other studies have associated empathy with lower levels of burnout and secondary stress trauma (Wagaman et al., 2015).

Numerous protective variables have been identified in recent literature related to counseling and traumatology that could be utilized as proactive coping strategies or as intervention coping strategies to lower the risk of stress, burnout, secondary traumatic disorder, neglectful self-care, compassion fatigue, and emotional and physical problems associated with the work environment. Active engagement in strategic self-care interventions, work-life balance, high level of social support, increased sense of accomplishment, and mitigating stress-related outcomes are examples of factors identified as protective factors against stressors come from work (Conrad and Kellar-Guenther, 2006; Alkema et al., 2008; Bourassa, 2009; Diaconescu, 2015; Salloum et al., 2015). The literature on the field of traumatology has also shown that the experience of compassion satisfaction is exceptionally significant as a protective factor to be used to better deal with work stressors and redirect them toward positive outcomes, such as enhancement in work performance, engagement, and competency (Radey and Figley, 2007; Snyder and Cistulli, 2009; Sacco and Copel, 2018; Raimondi, 2019).

Compassion satisfaction refers to the joy and positive emotions experienced by helping professionals from having the ability to complete their work effectively. For instance, they might consider it rewarding to have the capability to assist others through their work. They may have optimistic feelings about their coworkers, their ability to improve the working environment, or even the whole society (Stamm, 2010). Compassion satisfaction and its role in reducing compassion fatigue and other work-related stressors are well-researched in other helping and caregiving professions (Kraus, 2005; Carmel and Friedlander, 2009; Harr and Moore, 2011; Ray et al., 2013; Thomas, 2013: Wagaman et al., 2015; Pelon, 2017). However, research on compassion satisfaction among educators in higher education in general and among social work educators, in particular, is still rare, specifically research that focuses on strategies to encounter stress, compassion fatigue, burnout, and other adverse psychical and psychological outcomes associated with working as social work educators in higher education (Raimondi, 2019; Velez-Cruz and Holstun, 2022).

Although social work educators differ in job responsibilities from helping and caregiving professionals, social work educators in higher education could arguably experience compassion satisfaction. Increased knowledge about the experience of compassion satisfaction among this population may yield valuable information to inform interventions intended to counter the negative impacts on the professional quality of life associated with social work academia.

Thus, this study was conducted using qualitative research methodology to explore the phenomenon of compassion satisfaction among social work educators in higher education. Aims for this study included: (1) Identifying and describing the psychological essence of compassion satisfaction among social work educators working in higher education. (2) Investigating how social work educators engage in compassion satisfaction. (3) Understanding threats to compassion satisfaction among this population.

## Materials and methodology

The researchers used the method of phenomenology to explore, describe, understand, and interpret the lived experiences of social work educators. The subjective understanding of phenomena experienced by individuals becomes an integral and central part of the study findings (Moustakas, 1994). Thus, phenomenology allowed researchers to gain deep insight into the meaning-making processes (Qutoshi, 2018) associated with CS among this population. In addition, using the phenomenological approach afforded researchers opportunities to mine rich and descriptive narratives from the social work educators in the study (Moustakas, 1994). Throughout the research process and subsequent analysis, the researchers also used constructivist grounded theory analytic techniques (Charmaz, 2014) to aid in the meaning-making process of social work educators and discover strategies related to how they engaged in the phenomena of CS. Hence, there is an interplay in the phenomenological data collection with grounded theory analysis for this study (Lincoln and Guba, 1985; Spinelli, 1989; Moustakas, 1994; Charmaz, 2014).

Recruitment of participants included a convenience sample of social work educators in higher education from three different states. The final sample included 11 participants (*N* = 11). 100% of the individuals who agreed to participate in the study met the inclusion criteria and completed the study. The participants were not compensated for their participation. The study included only those who were actively teaching social work courses in higher education when the interviews were conducted. Participants’ employment status ranged from teaching one class to full-time. Sampling continued until data saturation was reached. For example, the research team discontinued recruitment when there was enough information to replicate the study, there was no ability to obtain new information, and further coding was no longer feasible (Guest et al., 2006). The researchers reached a repetition point, completed the codebook with no further codes needed, and enough data was collected for study replication.

This study was approved by the Institutional Review Board at [blinded for review]. Participants signed informed consent documents before participation and were allowed to ask clarifying questions to ensure their complete understanding of the consent process. To protect the confidentiality, the researchers asked all participants to provide a codename/pseudonym to be used throughout the analysis and reporting of the study’s findings. Respondents completed a demographic survey and *The Professional Quality of Life (ProQOL) Version 5 measurement instrument* (Stamm, 2010). In addition, the researchers used a semi-structured interview guide when conducting in-depth interviews. The researchers developed an interview guide based on the theoretical sensitizing concepts of CS, symbolic interactionism, and pragmatism. Thus, the researchers focused on the meaning participants provided CS as they had experienced it in their own lives.

The researchers conducted in-depth interviews in various locations, such as office spaces and public coffee shops, ranging from 20 to 45 min. The researchers engaged in careful listening, critical thinking, and asking additional probing questions, which are significant for gaining in-depth insight into the lived experience of compassion satisfaction among social work educators. Participant interviews were recorded and transcribed. Based on the constant comparative technique, each line of every transcript was assessed line by line via open coding (Boyatzis, 1998; Strauss and Corbin, 1998). The line-by-line coding was then used to create focused codes. Using the most significant and frequent codes, a codebook was developed. The thematic codes were then entered into Dedoose software. To reinforce the trustworthiness of study findings, the researchers used triangulation, with peer-debriefing and member checking of emergent themes as well as the use of multiple sources of data, such as field notes and participant scores from the Professional Quality of Life (ProQOL) Version 5 measurement instrument (Creswell and Miller, 2000; MacMillan and Koenig, 2004; Padgett, 2008; Stamm, 2010).

Furthermore, a situational analysis was utilized as an extension of grounded theory analysis techniques (Charmaz, 2014). Thus, messy, ordered, and positional maps were used in the data analysis (Clarke et al., 2018). To further the analysis, field notes, and analytical memos were utilized (Charmaz, 2014). Using Dedoose software, the researchers calculated intercoder agreement with a Kappa score of 0.92, considered an excellent agreement (Cohen, 1968). In addition, the researchers completed an adjudication process to look at test results to identify places where there were code disagreements. This iterative process helped to identify and edit segments in which codes disagreed, resulting in more accurately coded materials (Guest and MacQueen, 2008).

The experiences of the researchers as social work students and educators in higher education assisted them to acknowledge similar experiences and connect with the participants emotionally. This connection allowed them for deep and reflective responses during the research process. To reduce their subjectivity and minimize bias, the researchers engaged in bracketing, used field notes, sought consultation, and maintained inter-related and personal thoughts separately from that of participants through data collection and analysis (Moustakas, 1994; Maxwell, 2012; Tufford and Newman, 2012).

## Results

The sample for this study was comprised of participants who were mostly Caucasian females, with only two who self-identified as male. Only one participant self-identified as an African American. The mean age for the participants was 51 years old. The participants came from several different, mid-sized cities in the United States. Most participants (60%) held a master’s degree level of education, whereas 40% were PhDs. Five served as tenure track faculty, three in term positions, and three as adjunct faculty. The number of years participants worked as social work educators in higher education ranged from one to 30, with a mean time of 11 years. In addition, 64% of participants were not practicing social workers. The number of years that participants had practiced social work also varied, with a range of one to 60. The mean time participants had worked in social work practice social was 18 years (See Table 1).

**Table 1:** Participant demographics.

Codename	Highest Education	Current Position	Years as an educator	Currently Practicing	Years as a practitioner
Leigh	Doctoral	Professor	11–20	No	1–20
Cal	Master’s	Adjunct	11–20	No	21–40
Emily	Master’s	Instructor	1–10	Yes	1–20
Theresa	Doctoral	Professor	21–30	No	1–20
Chris	Doctoral	Assistant Professor	1–10	No	21–40
Samantha	Master’s	Instructor	1–10	No	21–40
Michael	Master’s	Instructor	11–20	No	41–60
Mary	Doctoral	Associate Professor	21–30	No	21–40
Mariah	Doctoral	Professor	21–30	Yes	21–40
Janet	Master’s	Adjunct	1–10	Yes	1–20
Bradford	Master’s	Adjunct	1–10	Yes	21–40

All participants who completed the Professional Quality of Life (ProQOL) Version 5 measurement instrument (Stamm, 2010) scored high on the Compassion Satisfaction scale. 77% of participants scored low on the Burnout scale, with only two having average scores. 88% scored low on the Secondary Traumatic Stress Scale, with only one participant scoring average. Within the context of social work education, participants described the experience of CS and threats to CS which encompassed four different elements: achievement, support, balance, and empathy. CS was experienced in two different realms: one’s educational setting, and one’s personal life. The researchers also identified several strategies utilized by social work educators to preserve CS and counter burnout and CF (See Table 2).

**Table 2:** Elements, realms, and strategies of compassion satisfaction for social work educators working in higher education.

Elements of CS	Realm	Strategies
1. Achievement	Educational	Perceiving student success Participating in energizing engagement
	Personal	Awareness of doing one’s best
2. Support	Educational	Giving and receiving collegial encouragement
	Personal	Fellowshipping with family and friends
3. Balance	Educational	Boundaries with Students
	Personal	Self-care
4. Empathy	Educational	Considering context of students’ personal lives
	Personal	Empathy for self

While the phenomenon of CS has mainly been studied among practitioners working with clients, the participants in this study were able to apply it to the educational realm and described how they experience CS. Two themes emerged: the impact on students and the students’ impact on clients. For example, Theresa described how she experienced CS by recognizing her ability to have an impact on students,

So as an educator, I think compassion satisfaction would be knowing you can help your students, motivate your students, and help your students get through some of the issues that might lead to compassion fatigue, to help them, to create awareness for them, and maybe some building blocks or tools to help themselves.

Additionally, Samantha explicated how she experienced CS thru recognizing the size of the students’ impact on clients, “I think of the thousands and thousands of clients they are going to interact with and how much of a difference I’m making through that and that’s huge.”

### Threats

Threats to CS identified by social work educators included bureaucracy, work overload, online teaching, and organizational culture. Participants named bureaucracy as the most frequent threat interfering with their experiences of CS. For example, Mariah explained the negative impact of institutional barriers on her experience as a social work educator, “Well, I have moments when I’m really ticked off and I’ve struggled with resentment over institutional barriers more than anything. It’s not the student.” Moreover, Mary explicated how the interaction with administrators and colleagues interfered with her experience of CS, “It’s interacting with administration and colleagues that gives me fatigue. It’s the system.” Participants also noted that the type of work done, as well as being overloaded posed threats to their experiences of CS. For example, Chris noted, “The work as a social work educator, I swear, you could just do nothing but work all the time, you really could, around the clock, um, there’s always something to do.” However, Cal addressed the difficulty in sustaining compassion satisfaction while teaching online courses, “Those are much harder for me to maintain that compassion satisfaction, because it’s really hard to develop relationships online. I struggle with that.” Finally, Leigh talked about the potentially negative impact the organizational culture of research-based institutions of higher learning might have on their educators when she voiced, “They’re really living and dying by that publish or perish, then I think it takes the educator sometimes away from what that primary purpose of being an educator is, and I think when you get pulled away from the primary purpose, whether you realize it or not, there is a, uh, a frustration, there’s a friction.”

### Coping strategies

The strategies participants most frequently mentioned related to experiencing CS concerned the element of achievement. These took place in both the educational realm and the personal realm. Within the personal realm, participants noted the strategies of perceiving student success and participating in energizing engagement. Student success was an important part of CS for many participants. For example, Mariah shared how an experience of CS was facilitated for her by recognizing the achievement of her students, as practitioners,

“I’ve just seen people that I’ve taught become outstanding clinicians, so that’s just wonderful. I did not cause that, but I had something to do with that, and that feels good.

Other participants saw achievements such as high-quality classroom engagement, which energized their work roles and lead to maintaining CS. Leigh noted, “I experience it when we get in a classroom discussion that just goes off the chain, and its, you know, phenomenally more than you could have hoped for…. there’s just something so satisfying and energizing when you come out of that experience.”

Within the personal realm, achievement was focused on self-awareness, such as realizing that one did the best job they possibly could have done. For example, Mary noted,

You cannot be dependent on some outside factor or some person constantly giving you, ‘Good job,’ ‘Thank you,’ all the time, because it’s not going to come. It needs to come from inside yourself, and that’s why it makes sense. You’re the compassion satisfaction or the … meaning you get from being able to help somebody else.

Likewise, Mariah echoed these sentiments when she said, “I do the best I can. I work on that; I try to work on integrity. I try to be who I am when nobody’s looking.” Self-awareness and self-reflection were related to a lack of support. Participants reported needing to develop these attributes within the personal realm if they were not receiving support from others in the work environment.

Many participants put forth narratives about collegial support as a strategy for experiencing CS within the educational realm. For instance, when she first began working in higher education, Theresa recalled a professor who, “…took me under their wing and really helped me, so I was glad that that person was there.” Likewise, Cal expanded on Theresa’s insights when she said, she had a full-time faculty lead that acted as “…kind of my liaison. She’s been very positive, you know, very full of compliments when I’m doing something well.” Participants also detailed that support also arose out of the personal realm and involved family and friends. For example, Mariah noted, “I have a strong network of friends and family and colleagues. All those things help.”

Most participants noted to achieve balance, setting boundaries was an important aspect of bolstering experiences with CS. The boundaries were described by participants as keeping appropriate lines between themselves as faculty and the students. This definition encompassed ideas of their roles as helpers, classroom work, and internal boundaries. Emily discussed the importance of having clear boundaries when she stated, “I will mentor them, talk to them kind of thing but if they full on need therapy, I will refer them out.” And, related to Emily’s sentiments on the topic, Theresa offered, “… my job is to help coach them, teach them, and have them learn. I see myself as being responsible to identify the barriers to their learning and get them the resources to help them with that.”

The participants also discussed balance as it related to feedback, grading, and fairness. Cal explained, “I get that students get frustrated, but I’m always very careful to phrase my feedback in a way that lets students own their behaviors and their mistakes.” Adding to that, Bradford put forth “…when helping a student, I try to regulate my ability to answer that and make sure I can do that for everybody. So, I make sure there is fairness and equity.”

Participants also noted that keeping good boundaries also meant internal boundaries. For example, Samantha explained that she does not take students’ challenging behavior personally when she elaborated, “I do not look at their performance that I’m responsible for, because there are too many other things going on in their life that I cannot intervene with.” Adding to that, Michael detailed: “One of the saddest days for me in social work, way long time ago, was learning that I could do the best job I could possibly do, and have a bad outcome, because I’m not in charge of the outcome.” Within the personal realm, self-care was a strategy that participants utilized to maintain balance and experience CS. Emily noted, “So, I guess if I take care of myself then I will be in a better mindset of things, and so, it’d be easier to deal with something that comes up.” Similarly, Michael noted, “We need to take more responsibility for our own outcomes, our own satisfaction index. I think we should pay more attention to how we are doing mentally, physically.”

Speaking to keeping boundaries as a form of self-care, Chris noted a strategy of being ‘done” when ‘the keys go in the basket” This shows a personal boundary between work and home. Adding to that, Chris vocalized: “It took me a while to, you know, work through, this profession…does not define you. And, once I worked my way through that, man, my compassion satisfaction rose… I just really engage in, you know, good self-care habits.”

Empathy came up when speaking about students, as well as empathy for themselves. Within the educational realm, the participants noted having extra compassion for students because of their personal challenges outside of the classroom. Mariah voiced.

It’s hard and grueling when you are a student. And I think part of compassion is understanding there is this whole range of what people bring to the room, and you often do not know what that is.

Empathy for self was noted in the personal realm. This appeared to not only allow a route to self-care, but also allow an honesty from students. As Bradford one day in her class she said to her students, “we are going to end class early, and we’ll pick it up next week, I only got three hours of sleep, I know I’ll be in a better place, and I’ll be able to convey these things to you in a much better way than I am right now, so I’m honest with them about my own life struggles, so they kind of know it’s okay to be honest about theirs.”

## Discussion

Current research in the field of traumatology identified CS as an essential protective factor against work-related stressors, such as burnout, compassion fatigue, and secondary traumatic disorder, emphasizing how CS leads to experiencing positive outcomes from work like improving work performance, increasing the level of engagement with clients, and enhancing competency (Radey and Figley, 2007; Snyder and Cistulli, 2009; Sacco and Copel, 2018; Raimondi, 2019). While stress, compassion fatigue, burnout, and other adverse psychical and psychological outcomes have been associated with working as social work educators in higher education (Stoves, 2014; Cordaro, 2020), research on CS among this population is still rare (Raimondi, 2019; Velez-Cruz and Holstun, 2022). In this study, we have acquired qualitative data from 11 social work educators in higher education to help elucidate the significance of CS from an emic perspective. This study addressed how the psychological essence of CS is identified and described among social work educators in higher education, explored how social work educators engage in compassion satisfaction, and unearthed a deeper understanding of threats to CS among this population.

A substantial number of participants were able to apply CS to the educational realm, indicating it can be experienced thru recognizing their abilities to have a positive impact on students and realizing the great size of the influence that students will have on clients in the future. This finding is consistent with how Stamm (2010) defined the experience of CS among helping professionals, the pleasure, positive emotions, and optimistic feelings experienced by those working in helping professions thru having the ability to complete their work effectively and assist others through their work.

A number of the participants in this study identified bureaucracy, overload, online teaching, and organizational culture as threats to CS. This is clearly seen in the challenges and struggles they experienced from institutional barriers, interaction with administrators and colleagues, work overload, online teaching, and involvement with the organizational culture of research-based institutions of higher learning, which interfered with their experiences of CS. These threats to CS demonstrate how work demands with a lack of administrative and collegial support in higher education institutions could produce adverse negative outcomes on this population’s professional quality of life and increase the likelihood of stress and burnout among this population (Hoffman et al., 2007; Kim and Stoner, 2008; Watts and Robertson, 2011; Chen, 2022).

Many of our participants focused on achievement, support, balance, and empathy as elements within the educational and personal realms encompassed their lived experience of compassion satisfaction. These four elements were used by participants in this study as practical tools for everyday life to cope with stressors that come from working as social work educators and to facilitate the experience of CS. Participants in this study spoke of a sense of achievement from perceiving students’ success, participating in energizing engagement, being a part of a high-quality classroom, and realizing that they did the best job they possibly could have done, which they used as an important coping strategy to deal with stress and experience CS. Numerous studies point to the significance of a sense of achievement as a protective factor against stress and compassion fatigue, which negatively influence the experience of CS (Bourassa, 2009; Harr and Moore, 2011; Kawar et al., 2019).

Participants in this study called upon support as a strategy for dealing with work stressors and experiencing CS. This is clearly seen in the collegial support they experienced within the educational realm and the family and friend support experienced within the personal realm, which contributed positively to their experience of CS as educators. As Drury et al. (2014) have shown, the capacity for coping with stress, burnout, and compassion fatigue can be enhanced through strong social and collegial support, which facilitates the experience of CS (Drury et al., 2014). In a recent study Yu and Gui (2022) point to the importance of perceived social support to improve CS and protect self against compassion fatigue (Way and Tracy, 2012; Yu and Gui, 2022).

The importance of achieving balance for the social work educators in higher education in this study was a significant tool to alleviate stress and experience CS. Setting and keeping good boundaries, balance as it related to feedback and personal boundary between work and home were some of the examples that participants in this study discussed as an important strategy for bolstering their experiences with CS. This finding reinforces Bae et al. (2020) when they indicated that work-life balance is associated with an increase in compassion satisfaction. The importance of self-care, which was discussed by participants in this study as a coping strategy to encounter compassion fatigue and experience CS is in line with past research (Conrad and Kellar-Guenther, 2006; Alkema et al., 2008; Harr et al., 2014; Salloum et al., 2015).

Our participants in this study also point to empathy as an important strategy to maintain CS, which is in line with the research showing that higher levels of empathy are associated with lower levels of burnout and secondary stress trauma (Wagaman et al., 2015). However, other studies have shown the possibility for a faculty member who exercises empathy and deeply cares for distressed students to become psychologically overwhelmed and develop compassion fatigue (Stoves, 2014; Cordaro, 2020).

## Future research recommendations

Stamm’s (2010) explications on CS among the helping professions and her concepts were helpful for analysis purposes. They appear to align well with the idea of CS as extended to social work educators. However, more research may help connect other aspects of CS to higher education. Implications for educators would be to attempt to use the strategies found to foster their own CS. Strategies that emerged could be beneficial for others to replicate, such as keeping good boundaries or finding a supportive person within their colleagues. Administrators in higher education should look carefully at the organizational culture of their institution, which may affect the CS of those in their educational departments. Thus, developing policies and procedures that assist social work educators in increasing self-care levels to sustain CS and encounter compassion fatigue and burnout.

## Limitations

Most of the participants in the study sample had taught for over 10 years, and this provided less data on those who were newer to the profession. Therefore, the results may not be as representative as those who are new educators. There were also more females (81%) than males (18%) represented in the participant sample. These percentages differed from the national average of 72.5% for females and 27% for males, as reported in the Council on Social Work Education’s (CSWE) 2017 annual report (CSWE, 2017). Thus, the sample may not be gender-representative of the overall social work educator population.

## Conclusion

This study provided a platform for participants to voice their lived experiences of CS within the educator domain. Based on the study’s findings, further research would be beneficial to further investigate each strategy found and the barriers within education to engaging in the strategy. Further, it would be interesting to investigate how CS differs for those teaching full-time versus those still practicing while working in higher education. Additional research could also focus on comparing CS within higher education across different disciplines to see if there are challenges or strategies unique to the social work education realm. Overall, the lived experiences of CS are a topic ripe for investigation in this field and essential for the well-being of educators. The experience of higher education professionals in social work education is an essential function of personal and educational professional well-being for both those in academia and the future social workers they shape.

## Data availability statement

The raw data supporting the conclusions of this article will be made available by the authors, without undue reservation.

## Ethics statement

The studies involving humans were approved by the Institutional Review Board (IRB) at the University of Louisville. The studies were conducted in accordance with the local legislation and institutional requirements. The participants provided their written informed consent to participate in this study.

## Author contributions

SS performed the qualitative analysis, participated in the design, and edited both the first and final drafts of the manuscript. BM helped with the data’s design and analysis. JZ carried out the qualitative analysis and contributed to the study’s design. All authors contributed to the article, read, edited, and approved the submitted version.

## Conflict of interest

The authors declare that the research was conducted in the absence of any commercial or financial relationships that could be construed as a potential conflict of interest.

## Publisher’s note

All claims expressed in this article are solely those of the authors and do not necessarily represent those of their affiliated organizations, or those of the publisher, the editors and the reviewers. Any product that may be evaluated in this article, or claim that may be made by its manufacturer, is not guaranteed or endorsed by the publisher.
